# Radiomics Signatures of Cardiovascular Risk Factors in Cardiac MRI: Results From the UK Biobank

**DOI:** 10.3389/fcvm.2020.591368

**Published:** 2020-11-02

**Authors:** Irem Cetin, Zahra Raisi-Estabragh, Steffen E. Petersen, Sandy Napel, Stefan K. Piechnik, Stefan Neubauer, Miguel A. Gonzalez Ballester, Oscar Camara, Karim Lekadir

**Affiliations:** ^1^BCN MedTech, Department of Information and Communication Technologies, Universitat Pompeu Fabra, Barcelona, Spain; ^2^William Harvey Research Institute, NIHR Barts Biomedical Research Centre, Queen Mary University of London, London, United Kingdom; ^3^Barts Heart Centre, St. Bartholomew's Hospital, Barts Health NHS Trust, London, United Kingdom; ^4^Department of Radiology, Stanford University, Stanford, CA, United States; ^5^Division of Cardiovascular Medicine, Radcliffe Department of Medicine, University of Oxford, Oxford, United Kingdom; ^6^Catalan Institution for Research and Advanced Studies (ICREA), Barcelona, Spain; ^7^Departament de Matematiques i Informatica, Universitat de Barcelona, Artificial Intelligence in Medicine Lab (BCN-AIM), Barcelona, Spain

**Keywords:** cardiovascular magnetic resonance, radiomics, machine learning, cardiovascular risk factors, UK biobank

## Abstract

Cardiovascular magnetic resonance (CMR) radiomics is a novel technique for advanced cardiac image phenotyping by analyzing multiple quantifiers of shape and tissue texture. In this paper, we assess, in the largest sample published to date, the performance of CMR radiomics models for identifying changes in cardiac structure and tissue texture due to cardiovascular risk factors. We evaluated five risk factor groups from the first 5,065 UK Biobank participants: hypertension (*n* = 1,394), diabetes (*n* = 243), high cholesterol (*n* = 779), current smoker (*n* = 320), and previous smoker (*n* = 1,394). Each group was randomly matched with an equal number of healthy comparators (without known cardiovascular disease or risk factors). Radiomics analysis was applied to short axis images of the left and right ventricles at end-diastole and end-systole, yielding a total of 684 features per study. Sequential forward feature selection in combination with machine learning (ML) algorithms (support vector machine, random forest, and logistic regression) were used to build radiomics signatures for each specific risk group. We evaluated the degree of separation achieved by the identified radiomics signatures using area under curve (AUC), receiver operating characteristic (ROC), and statistical testing. Logistic regression with L1-regularization was the optimal ML model. Compared to conventional imaging indices, radiomics signatures improved the discrimination of risk factor vs. healthy subgroups as assessed by AUC [diabetes: 0.80 vs. 0.70, hypertension: 0.72 vs. 0.69, high cholesterol: 0.71 vs. 0.65, current smoker: 0.68 vs. 0.65, previous smoker: 0.63 vs. 0.60]. Furthermore, we considered clinical interpretation of risk-specific radiomics signatures. For hypertensive individuals and previous smokers, the surface area to volume ratio was smaller in the risk factor vs. healthy subjects; perhaps reflecting a pattern of global concentric hypertrophy in these conditions. In the diabetes subgroup, the most discriminatory radiomics feature was the median intensity of the myocardium at end-systole, which suggests a global alteration at the myocardial tissue level. This study confirms the feasibility and potential of CMR radiomics for deeper image phenotyping of cardiovascular health and disease. We demonstrate such analysis may have utility beyond conventional CMR metrics for improved detection and understanding of the early effects of cardiovascular risk factors on cardiac structure and tissue.

## Introduction

Cardiovascular magnetic resonance (CMR) is the reference standard for assessment of cardiac structure and function and is used widely in both research and clinical settings. Routine assessment is reliant on visual inspection of CMR images for identifying global and local abnormalities; this is both labor-intensive and reader dependent (1–4). Existing quantifiers, such as ejection fraction and chamber volumes, are overly simplistic and often do not capture subtle and complex changes that affect the myocardium at early disease stages (5). Current approaches are thus suboptimal for early disease detection and outcome prediction. Therefore, there is need for novel, more advanced quantitative approaches to CMR image analysis to improve clinical diagnosis and risk prediction.

CMR radiomics is a novel image quantification technique whereby pixel-level data is analyzed to derive multiple quantifiers of tissue shape and texture (6). Technological advancements and the availability of high computational power has allowed deployment of machine learning (ML) methods with radiomics features to discriminate disease or predict outcomes (7). A distinct advantage of radiomics modeling over unsupervised algorithms is the potential for explainability through identification of the most defining radiomic features in the model. It is thought that radiomics features correspond to alterations at both the morphological and tissue levels and thus, the most defining features of a particular condition (or its radiomics signature) may provide insights into its pathophysiology (8). Within oncology, where radiomics is most well-developed, the incremental value of radiomics models for diagnosis and prognosis have been widely reported (8–14). In cardiology, early studies have shown promising results from CMR radiomics models for discrimination of important conditions such as myocarditis, hypertrophic cardiomyopathy, and ischemic heart disease (15–18).

While existing works have mostly focused on image phenotyping of established cardiovascular diseases, CMR radiomics may also provide incremental information to conventional approaches for improved quantification of cardiac alterations related to cardiovascular risk factors at the subclinical stage. We thus present the largest and most comprehensive assessment of the performance of CMR radiomics for image phenotyping of important cardiovascular risk factors including diabetes, hypertension, high cholesterol, and smoking status, by using a large annotated CMR dataset from the UK Biobank (UKB).

## Methods

### Population and Setting

UKB is a large-scale population health resource aimed at enhancing biomedical research and ultimately improving prevention, diagnosis, and treatment of a wide range of serious and life-threatening illnesses (19). Over 500,000 participants aged 40–69 years old were recruited from around the UK between 2006 and 2010. The UK Biobank holds an exceptional amount of data including detailed lifestyle information, medical history, serum biomarkers, physical measures, and multi-modal imaging including magnetic resonance imaging of the abdomen, brain, and heart (20). Thus, UKB provides the ideal platform for assessment of the performance characteristics of novel quantitative biomarkers, such as radiomics, in discriminating common cardiovascular risk factors.

### CMR Imaging Protocol

CMR cine images were acquired using a standardized UKB protocol, which is detailed in a dedicated publication (21). In brief, all scans were performed with a 1.5 Tesla scanner (MAGNETOM Area, Syngo Platform VD13A, Siemens Healthcare, Erlangen, Germany), with typical cine parameters as follows: TR/TE (repetition time/echo time) = 2.6/1.1 ms, flip angle 80°, Grappa factor 2, voxel size 1.8 × 1.8 × 8 mm, and a slice gap of 2.0 mm. The actual temporal resolution of 32 ms was interpolated to 50 phases per cardiac cycle (~20 ms). The protocol includes a complete cine short-axis ventricular stack with base to apex coverage acquired using balanced steady state free procession (bSSFP) with one breath-hold per image slice.

### CMR Image Segmentation

CMR scans of the first 5,065 UKB participants that completed the imaging study were manually analyzed across two core laboratories (London, Oxford) using a pre-defined standard operating procedure, which is detailed elsewhere (22). In brief, left and right ventricular (LV, RV) endocardial contours and LV epicardial contours were drawn in end-systole and end-diastole on the short axis stack images using the CVI42 post-processing software (Version 5.1.1, Circle Cardiovascular Imaging Inc., Calgary, Canada). These contours were used to define three regions of interest (ROIs) for radiomics analysis: RV blood pool, LV blood pool, and LV myocardium. All acquisitions were ECG gated and thus end-diastole was defined as the first phase in the sequence. End-systole was defined as the frame with smallest LV cavity area by visual assessment detected at the mid-cavity level. Papillary muscles were considered part of the blood pool. Slices with more than 50% circumferential LV myocardium were included in LV contours. RV volume was defined as areas below the pulmonary valve plane identified by visual assessment.

### Selection of Study Sample

**Figure 1:**
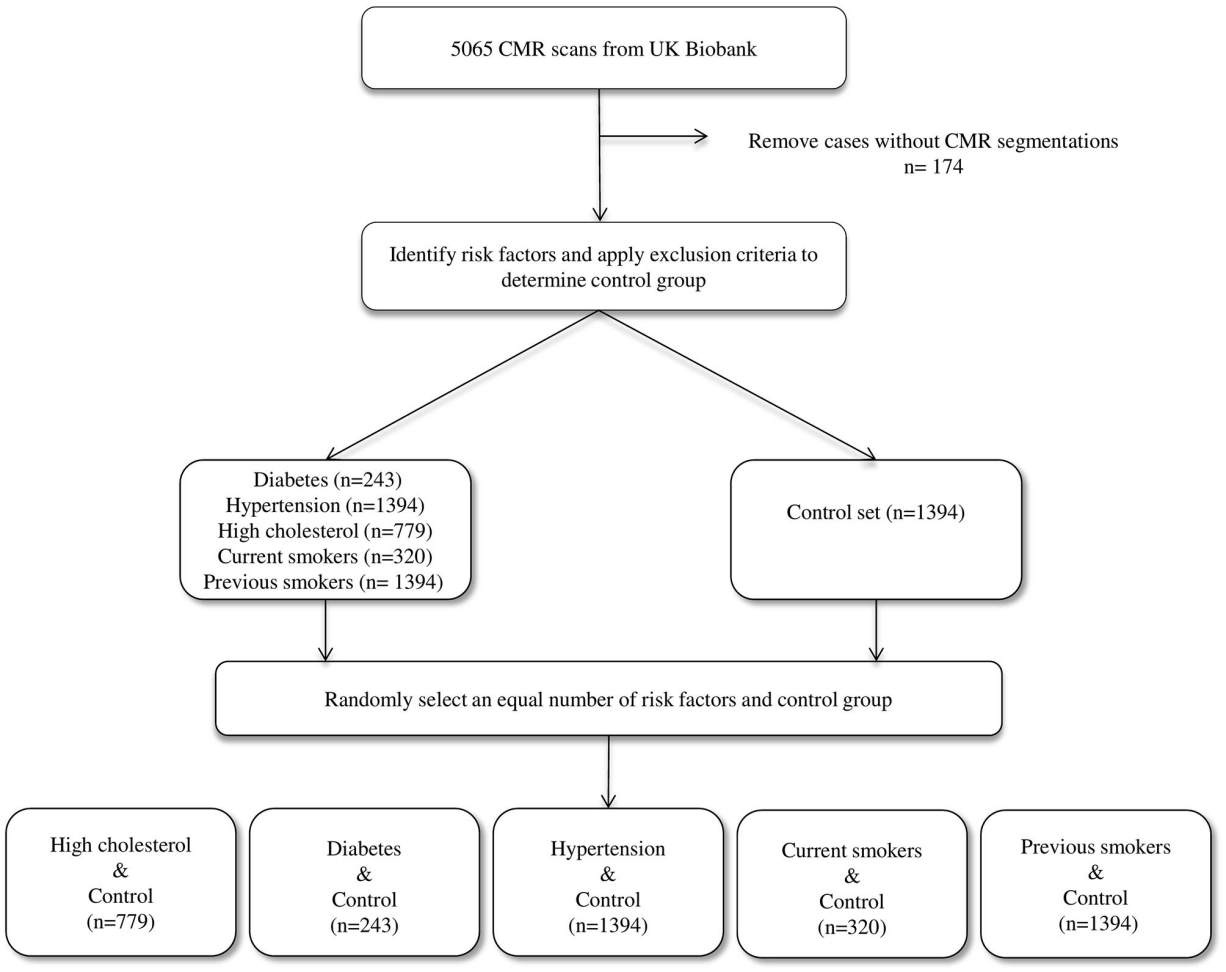
The data selection process.

We considered the first 5,065 UKB participants to complete CMR imaging. We excluded 174 individuals due to incomplete segmentations (having either one or more cardiac structures missing in the segmentations). From the remaining 4,891 individuals, a healthy cohort (*n* = 1,394) was defined by considering participants without known cardiovascular disease or risk factors. Diabetes (*n* = 224), hypertension (*n* = 1,394), and high cholesterol (*n* = 779) were taken from self-reported conditions. Smoking status was taken as self-report of current (*n* = 320) or previous (*n* = 1,394) tobacco smoking. Participants positive for each risk factor were compared with an equal number of randomly selected reference healthy subjects to eliminate bias in the machine learning models due to class imbalance (Figure 1).

### Conventional CMR Indices

For comparison and quantification of the added value of CMR radiomics, conventional CMR indices were also assessed, specifically: LV end-diastolic volume (LVEDV), LV end-systolic volume (LVESV), RV end-diastolic volume (RVEDV), RV end-systolic volume (RVESV), LV stroke volume (LVSV), RV stroke volume (RVSV), LV ejection fraction (LVEF), RV ejection fraction (RVEF), LV mass (LVM).

### Radiomics Analysis

**Figure 2:**
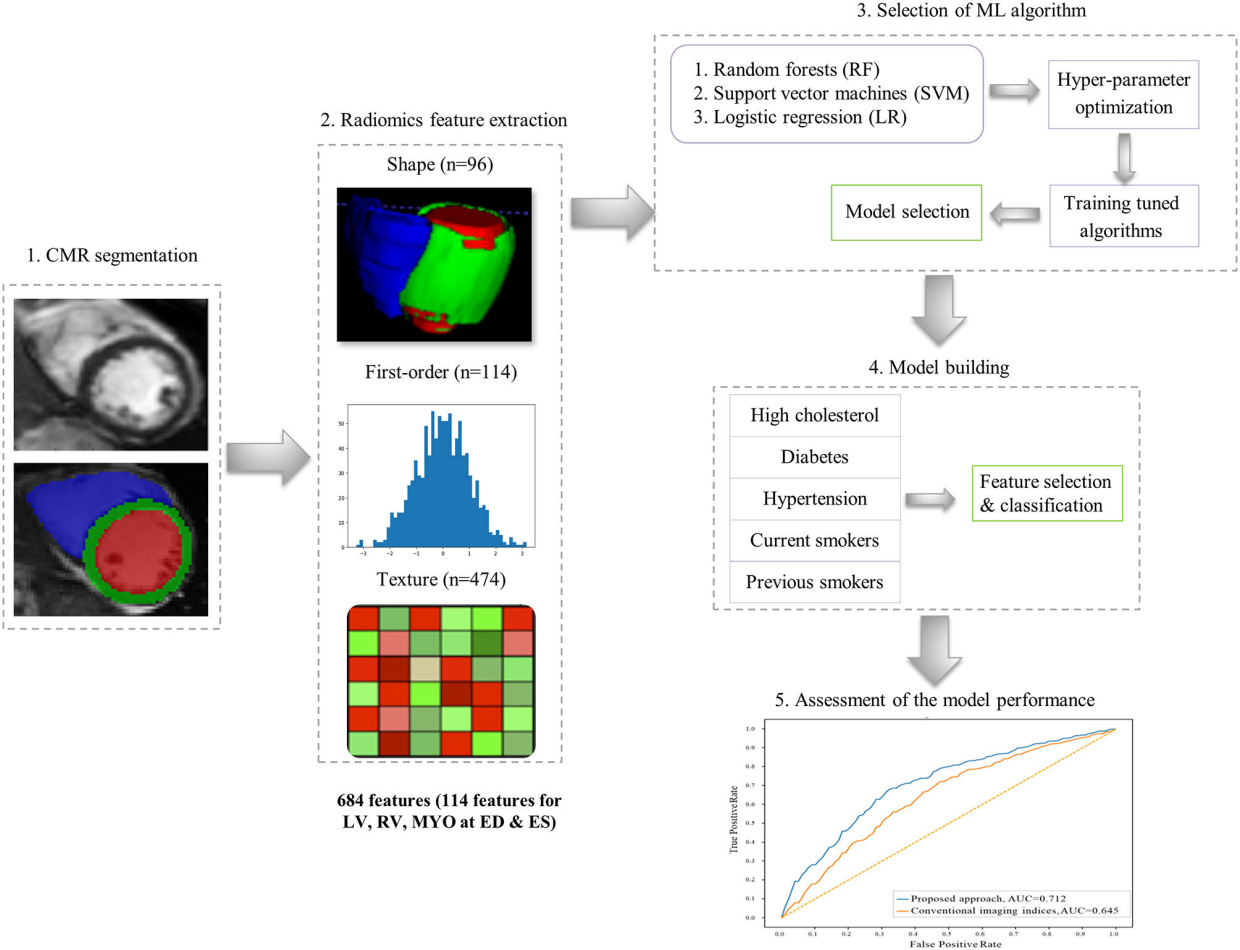
The proposed radiomics workflow.

The overall radiomics workflow is depicted in Figure 2. Radiomics shape and signal intensity-based features were extracted from the three segmented ROIs (LV blood pool: LV, LV myocardium: MYO, RV blood pool: RV) in end-diastole (ED) and end-systole (ES). The analysis of the radiomics features in the myocardium may enable identification of tissue-level changes due to the cardiovascular risk factors. The inclusion of the LV and RV cavities is aimed at identifying changes in the shapes of each ventricle, or in the patterns of the trabeculation and papillary muscles. Automated extraction of radiomics features was performed using the open source python-based radiomics library Pyradiomics (version 1.3.0, October 2017)^1^ (23). The customization of image preprocessing and feature extraction was performed with Pyradiomics default settings, including a gray value discretization with a bin width of 25 to extract the intensity-based and texture radiomics features. In total, 684 radiomics features were extracted per study (consisting of 114 radiomics features per cardiac structure: LV, RV and MYO at two time-points of the cardiac cycle: ED and ES).

#### Shape-Based Radiomics Features

16 radiomics shape features were extracted per ROI at ED and ES (see Supplementary Table). Radiomics shape features describe geometrical properties of the defined ROI, such as volume, maximal diameter, minor/major axis, surface area volume ratio, elongation, flatness, and sphericity. Radiomics shape features may provide incremental value to existing CMR indices as they include conventional shape indices (e.g., cavity volumes) as well as more advanced geometric quantifiers (e.g., sphericity, flatness). They also have the potential to define disease-specific patterns of cardiac alterations beyond those possible with existing CMR indices.

#### Signal Intensity-Based Radiomics Features

Signal intensity-based radiomics features may have the potential to decode variations in cardiac tissue due to abnormalities induced by disease processes. They are commonly grouped into two categories, namely first-order and texture features. First-order features are histogram-based statistics describing the global distribution of signal intensities within the defined ROI without consideration to their spatial relationships. These include simple measures such as the mean intensity or standard deviation, as well as more advanced measures such as skewness, uniformity or entropy (see full list in Supplementary Table).

#### Texture-Based Radiomics Features

In contrast, texture radiomic features allow the quantification of spatial inter-pixel relationships using more advanced matrix analysis methods (24, 25). Through this, signal intensities patterns within the ROI may be numerically quantified using pre-agreed mathematical definitions. Many texture patterns may be considered to quantify characteristics such as the complexity, heterogeneity, coarseness, or repeatability of the building blocks of the tissue. The idea is that these texture features may reflect myocardial tissue characteristics which in turn reflect underlying disease processes. In this study, 19 first-order features and 79 texture features were extracted from each ROI per cardiac phase.

### Identification of Optimal Radiomic Signatures

The goal of the study is to leverage feature selection and machine learning techniques to identify radiomics signatures that best describe the structural and tissue differences between risk factor (at-risk) and healthy (no-risk) groups in CMR imaging. To this end, we use the risk factors as “proxy” output variables and build multiple machine learning models by varying the combinations of input radiomic features through systematic feature selection. We obtain multiple models (and thus multiple candidate radiomic signatures) and through statistical testing one can select the best model and therefore the radiomic signature that best separate the at-risk and no-risk groups. Because these selected radiomics signatures differentiate at-risk from healthy individuals, they can be considered and analyzed as potential descriptors of the cardiac alterations due to the risk factors in question. Importantly, we use machine learning as a more advanced means to combine multiple radiomic features into risk-specific signatures, while taking into account non-linear complementarities between the parameters.

For feature selection, we used the sequential forward feature selection (SFFS) method as it has demonstrated good performance in previous CMR radiomics studies (15, 26). The termination criterion was set to 2% in all experiments following literature standards, i.e., the process was stopped if an added feature did not increase model performance beyond the termination criterion. To obtain more robust estimates and improve generalizability, ten-fold cross-validation was used in the feature selection process, rotating training and validation folds (80 and 20% of the dataset, respectively). We combined SFFS with classical ML algorithms [support vector machines (SVM), random forests (RF), logistic regression (LR)] to identify the combination of radiomics features that best define each studied cardiovascular risks/subgroups. For each ML method, hyperparameter optimization was performed to enhance the discrimination between no-risk and at-risk subgroups (Supplementary Material). Implementation of the SFFS and the ML techniques was based on the mlxtend (version 0.17.0) (27) and scikit-learn (version 0.20.3) (28) python-based libraries, respectively.

The selected radiomics features resulting from the SFFS algorithm and ML techniques were combined to create the radiomics signature that best encode the changes in CMR induced by the different cardiovascular risk factors. To quantify the added value of the proposed radiomics approach, we built similar ML models/risk signatures using conventional CMR indices as input variables. Note that all radiomics features and cardiac indices were normalized (to a mean of zero and standard deviation of one) to ensure they are equally weighted in all analyses. Note that individuals with multiple risk factors were not excluded. In the machine learning models, we set the outcome to each risk factor individually, which enabled the identification of the radiomics signatures specific to that risk factor.

In this work, we assess model performance (i.e., the ability of the radiomics signatures to discriminate at-risk vs. no-risk subjects) using receiver operating characteristic (ROC) curve and area under the curve (AUC) scores. We also report model accuracy, defined as number of correctly discriminated no-risk vs. at-risk cases based on the radiomics signatures, divided by the total number of cases. Additionally, statistical tests were performed to assess the statistical significance of the differences between the various ML models, by using the McNemar's test for pairwise comparisons, as well as the Cochran's Q test, which is an extension of the McNemar's test for the comparison of more than two models (29, 30).

## Results

### Summary of Subgroups and Conventional CMR Indices

The subjects included in the analysis are summarized in Table 1. Across all risk factor groups there was higher proportion of male participants (between 52.3 and 60.1% depending on the risk factor), whereas in the healthy cohort, there were fewer men (42.5%). Average age across the risk groups was between 59 (±8) and 65 (±6) years, while it was equal to 60 (±7) years for the healthy cohort. As expected, there were differences in conventional CMR between the at-risk subgroups and healthy subjects. In particular, all risk groups had on average greater indexed left ventricle mass (LVMi) in comparison to the healthy cohort with the greatest difference in the hypertensive group (50.3 g/m^2^ vs. 46.3 g/m^2^). All risk factor groups had lower indexed left ventricle stroke volume (LVSVi) and indexed right ventricle stroke volume (RVSVi) in comparison to the healthy cohort. There were also variations in chamber volumes, with different directions of difference depending on the risk category. Finally, it is worth noting that no statistically significant differences (Welch's *t*-test) in the conventional indices were found between the healthy and each at-risk subgroups, except for LVEF in diabetes and LVSVi values in hypertension and current smokers (see Table 1).

**Table 1:** Summary of conventional CMR indices for the risk and healthy groups included in the analysis.

	**Diabetes**	**Hypertension**	**High cholesterol**	**Current smoker**	**Previous smoker**	**Healthy**
	*n* = 243	*n* = 1,394	*n* = 779	*n* = 320	*n* = 1,394	*n* = 1,394
Male *n*(%)	146 (60.1%)	786 (56.4%)	460 (59.1%)	172 (53.8%)	729 (52.3%)	592 (42.5%)
Age mean(sd)years	64 (±7)	64 (±7)	65 (±6)	59 (±8)	63 (±7)	60 (±7)
LVEDVi (ml/m^2^)	73.4 (±13.8)	76.7 (±14.2)	75.0 (±13.9)	77.2 (±15.1)	76.9 (±14.8)	77.9 (±14.7)
LVESVi (ml/m^2^)	30.8 (±9.2)	31.6 (±9.3)	30.8 (±8.8)	32.5 (±9,4)	31.9 (±10.5)	31.6 (±8.8)
LVMi (g/m^2^)	49.1 (±9.6)	50.3 (±10.2)	48.6 (±9.7)	49.3 (±9.9)	48.3 (±10.1)	46.3 (±9.7)
LVEF (%)	58.5 (±7.3)^*^	59.2 (±6.9)	59.3 (±6.7)	58.3 (±6.9)	59.0 (±6.7)	59.7 (±5.9)
LVSVi (ml/m^2^)	42.7 (±8.3)	45.2 (±8.4)^*^	44.2 (±8.3)	44.7 (±8.9)^*^	45.1 (±8.2)	46.3 (±8.8)
RVEDVi (ml/m^2^)	77.2 (±14.5)	80.1 (±14.9)	79.1 (±14.9)	81.2 (±16.1)	80.8 (±14.8)	83.1 (±16.2)
RVESVi (ml/m^2^)	34.3 (±9.6)	34.8 (±9.7)	34.7 (±9.7)	36.3 (±10.4)	35.6 (±9.5)	36.8 (±10.5)
RVEF (%)	56.0 (±6.9)	56.9 (±6.7)	56.5 (±6.8)	55.7 (±6.9)	56.3 (±6.4)	56.2 (±6.3)
RVSVi (ml/m^2^)	42.9 (±8.2)	45.3 (±8.4)	44.4 (±8.5)	44.9 (±8.9)	45.2 (±8.3)	46.3 (±8.5)

LV, left ventricle; RV, right ventricle; EDV, end-diastolic volume; ESV, end-systolic volume; SV, stroke volume; EF, ejection fraction; LVM, left ventricle mass; i, indexed; absolute values divided by body surface area (calculated according to Du Bois formula). Values are given as mean ± standard deviation for continuous variables; and count (%) for categorical variables.

* *Indicates statistical differences with respect to the healthy subgroup according to Welch's t-test*.

### Radiomics Signatures Have Superior Discriminatory Performance Over Conventional CMR Indices

**Figure 3:**
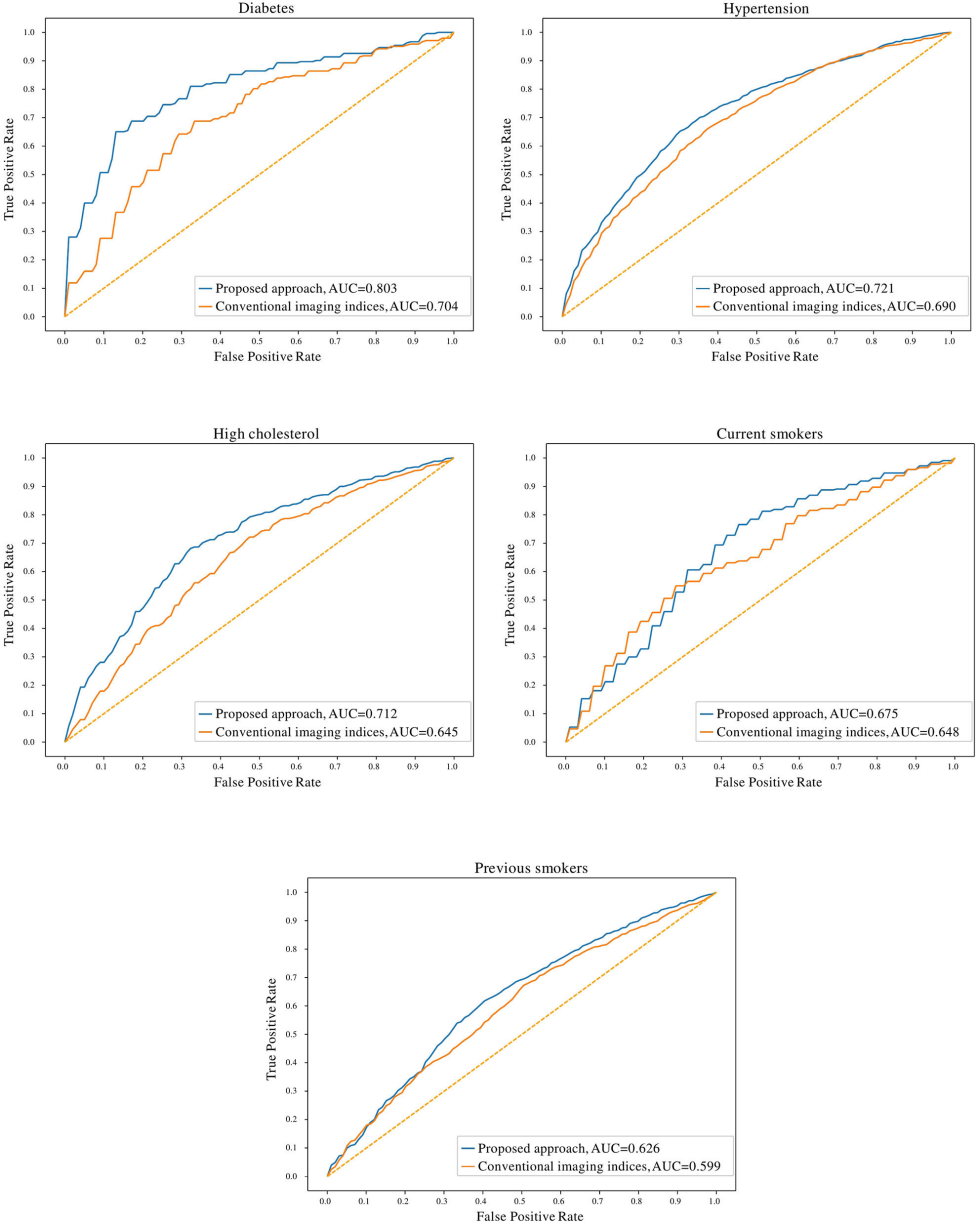
Receiver operating characteristic curves for radiomics and conventional CMR indices models for the cardiovascular risk factor subgroups. AUC: area under the curve.

In comparison to conventional indices, radiomics signatures provided better discrimination between healthy and at-risk subjects for diabetes (0.80 AUC for radiomics vs. 0.70 for conventional indices), hypertension (0.72 vs. 0.69), high cholesterol (0.71 vs. 0.65), and previous smokers (0.63 vs. 0.60) (Figure 3). The obtained models with radiomics vs. conventional indices were also compared using the McNemar's test; the differences were found to be statistically significant for diabetes, hypertension, high cholesterol, and previous smokers but not for current smokers.

### Comparison of the Degree of Discrimination Achieved for Each Subgroup

The degree of discrimination (no-risk vs. at-risk hearts) achieved using radiomics models varied between the different cardiovascular risks, as these have different effects on the heart. The highest degree of discrimination with radiomics models was seen in diabetes (0.78), suggesting that radiomics features are particularly important in distinguishing diabetes-related cardiac changes. The smallest degree of separation was seen in previous smokers (0.61). High cholesterol, hypertension and current smokers achieved similar degrees of separation by the radiomics models (i.e., 0.68, 0.68, and 0.67, respectively).

### The Identified Radiomics Signatures for Each Cardiovascular Risk Factor

The identified radiomics signatures for each risk factor are described in Table 2. Overall, there was a more prominent role for shape and texture features than first-order features. For instance, in diabetics, five of the eleven features included in the model were shape-based and in the hypertension group, no first-order feature was selected. As expected, radiomics features from the LV blood pool and LV myocardium were the most relevant regions, with the RV blood pool having a minor role for the risk factors studied in this paper.

**Table 2:** Radiomics features selected for each risk factor. Features are presented in order of importance (accuracy using only one feature) in the model for each risk factor.

**CV risk factor**	**Radiomics signature**	**Feature type**	**ROI**	**Phase**	**Alone**
High cholesterol	Spherical disproportion	Shape	MYO	ED	0.61
	Compactness	Shape	MYO	ED	0.60
	Skewness	First-order	LV	ED	0.59
	Informal measure of correlation	Texture	LV	ES	0.57
	Gray level non-uniformity	Texture	RV	ED	0.55
	Contrast	Texture	RV	ES	0.52
Diabetes	Median	First-order	MYO	ES	0.65
	Surface area to volume ratio	Shape	MYO	ED	0.61
	Energy	First-order	LV	ED	0.61
	Surface area	Shape	MYO	ES	0.58
	Dependence variance	Texture	LV	ED	0.57
	Large area high gray level emphasis	Texture	MYO	ED	0.57
	Energy	First-order	LV	ES	0.57
	Flatness	Shape	RV	ED	0.56
	Surface area	Shape	LV	ES	0.55
	Max 2D diameter column	Shape	RV	ED	0.50
	Difference average	Texture	LV	ES	0.44
Hypertension	Surface area to volume ratio	Shape	MYO	ED	0.61
	Percentile 10	First-order	RV	ES	0.58
	Informal measure of correlation	Texture	LV	ES	0.55
	Dependence non-uniformity normalized	Texture	LV	ED	0.54
	Size zone non-uniformity normalized	Texture	RV	ED	0.54
Current smokers	Gray level non-uniformity	Texture	MYO	ES	0.60
	Dependence entropy	Texture	LV	ED	0.57
	Standard deviation	First-order	MYO	ED	0.53
	Max 2D diameter column	Shape	RV	ED	0.50
	Large dependence low gray level emphasis	Texture	RV	ED	0.45
Previous smokers	Surface area to volume ratio	Shape	MYO	ED	0.57
	Busyness	Texture	LV	ES	0.54
	Run entropy	Texture	MYO	ES	0.50
	Skewness	First-order	RV	ES	0.50
	Run length non-uniformity	Texture	RV	ED	0.49
	Zone variance	Texture	LV	ED	0.49

*ROI, region of interest, Alone: model performance using each radiomic feature individually; LV, left-ventricle; RV, right-ventricle; MYO, left ventricle myocardium; ED, end-diastolic*.

In Table 3, we consider the most discriminative radiomics feature for each risk factor, i.e., the feature assigned the most importance in the model, and compare it with the most discriminative conventional CMR measure, which was LVM for all risk groups.

**Table 3:** Values of the best radiomics features (Rad) and the conventional CMR indices (Conv).

**CV risk factor**	**Single most defining feature**	**CV risk cohort**		**Healthy cohort**		**ACC**
		**Mean**	**SD**	**Mean**	**SD**	
High cholesterol	Rad: Spherical disproportion MYO ED (S)	3.631	0.290	3.779	0.311	0.611
	Conv: LVM (g)	93.493	24.199	85.667	24.104	0.576
Diabetes	Rad: Median MYO ES (F)	67.887	9.058	74.652	10.514	0.658
	Conv: LVM (g)	97.856	24.250	85.931	25.024	0.605
Hypertension	Rad: Surface area to volume ratio MYO ED (S)	0.390	0.054	0.425	0.06	0.618
	Conv: LVM (g)	97.131	25.849	85.623	24.101	0.593
Current smokers	Rad: Gray level non uniformity MYO ES (T)	573.448	134.355	515.789	140.307	0.609
	Conv: LVM (g)	93.614	24.804	84.549	25.426	0.564
Previous smokers	Rad: Surface area to volume ratio MYO ED (S)	0.405	0.058	0.425	0.062	0.574
	Conv: LVM (g)	91.902	24.896	85.623	24.101	0.552

*Feature values from risk groups and healthy individuals were statistically significantly different for all selected features (Bonferroni adjusted *p*-value < 0.05/684). S, shape; F, first-order; T, texture; SD, standard deviation; ACC, accuracy; CV, cardiovascular; MYO, LV myocardium; ED/ES, end-diastole/systole; LVM, left ventricular mass (in grams; g)*.

For all the subgroups, the mean value of the most important radiomics features and conventional CMR indices was significantly different in the risk factor vs. healthy cohorts (*p* < 0.001, Table 3). In addition, the single best radiomics feature outperformed the conventional CMR indices in its relevance for all risk factors. However, it was the combination of several radiomics features into a radiomic signature (Table 4) that provided the best overall discriminative power.

**Table 4:** Selected number of radiomic features used for each risk factor and their discriminative accuracy, and results obtained based on conventional imaging indices and size information.

**Risk factor**	**Radiomics features**					**Clinical indices**		
	**#**	**S/F/T**	**LV/RV/MYO**	**ED/ES**	**ACC/AUC**	**#**	**LV/RV**	**ACC/AUC**
High cholesterol	6	2/1/3	2/2/2	4/2	0.682/0.712	2	1/1	0.626/0.645
Diabetes	11	5/3/3	5/2/4	6/5	0.782/0.803	4	3/1	0.681/0.704
Hypertension	5	2/0/3	2/2/1	3/2	0.682/0.721	2	1/1	0.646/0.690
Current smokers	5	1/1/3	1/2/2	5/0	0.675/0.675	3	2/1	0.628/0.648
Previous smokers	6	1/1/4	2/2/2	3/3	0.612/0.626	2	1/1	0.579/0.599

*#, total selected number of features; S, shape features; F, first-order radiomics; T, texture features; LV, left ventricle; RV, right ventricle; MYO, Myocardium; ED, end-diastole; ES, end-systole; ACC, accuracy (prediction performance); AUC, area under the curve*.

## Discussion

### Summary of Findings

This paper described a methodology based on radiomics, machine learning and feature selection to discover new discriminatory signatures in CMR. Based on over 5,000 datasets, we presented the largest and most comprehensive study to demonstrate the feasibility and performance of CMR radiomics for identifying new imaging signatures associated with important cardiovascular risk factors such as diabetes, hypertension, cholesterol, and smoking. Over conventional indices, we showed that radiomics enable improved quantification of alterations in both cardiac structure and tissue due to the effects of these risk factors. From the statistical tests performed in Table 1, it can be seen that the conventional indices do not capture statistically significant differences between the healthy vs. at-risk subgroups, with very few exceptions (LVEF values in diabetes, LVSVi values in hypertension and current smokers). In contrast, the McNemar's statistical tests comparing the radiomics models and the conventional indices show statistically significant differences between the two approaches for all cardiovascular risk factors, except for current smokers. This indicates that for diabetes, hypertension, high cholesterol and previous smokers, radiomics models provide incremental value in identifying structural and textural differences between healthy and at-risk subgroups.

### Clinical Interpretation of the Radiomics Signatures

A distinct advantage of radiomics modeling over black-box techniques such as deep learning is the potential interpretability of the obtained results. Therefore, we can attempt to reason the prominence of certain radiomics features in disease discrimination models. Shape features were highly featured in all models and indicate subtle patterns of ventricular remodeling that are specific to conditions under study. For instance, spherical disproportion (i.e., the inverse of sphericity) of the myocardium at end-diastole was lower in participants with high cholesterol compared with healthy individuals, indicating that the overall shape of the LV is less elliptical and more spherical in this risk factor group. For hypertensive individuals and previous smokers, the surface area to volume ratio was smaller in the risk subgroups vs. healthy subjects; this may reflect a pattern of concentric LV hypertrophy in these conditions. For certain risk factors, intensity/texture features seemed more important, such as median intensity for diabetes. As this was a retrospective study, we can only speculate as to the cause of this association. One hypothesis is that diabetes leads to a global alteration of the myocardial tissue and thus of the overall myocardial appearance in CMR images, resulting in higher median intensities compared to non-diabetic subgroups. However, testing this hypothesis is beyond the scope of this study.

As another example of a prominent textural feature, the most important feature identified for current smokers in this study was gray level non uniformity. In a previous study (31), the same radiomic feature was identified as the most important radiomic feature in hypertrophic cardiomyopathy (HCM). However, as the authors pointed out in their paper, the intensity heterogeneity of myocardial tissue is not unique to HCM and it might be of importance for other conditions. As smoking is a well-known cause for such cardiovascular diseases (32), there may be some commonality in the patterns of myocardial hypertrophy and tissue fibrosis in these conditions that is being reflected in the observed texture features. Indeed, the increased heterogeneity in gray level intensities for current smokers as found in our study supports the potential effects on the myocardium for these subjects.

Thus, radiomics allows more granular distinctions between health and disease in comparison to conventional CMR indices where, rather crudely, the single most discriminatory feature for all risk factors was higher LVM. These findings indicate the potential clinical utility of radiomics in improving understanding of the effects and pathophysiology of important cardiovascular risk factors.

### Comparison With the Existing Literature

Literature in support of the superior diagnostic performance of CMR radiomics models over conventional image analysis is slowly gaining momentum. Several studies have shown the feasibility and clinical utility of CMR radiomics for distinguishing important disease entities. A small study by Baeßler et al. (31) demonstrates the superior performance of CMR radiomics in discriminating hypertrophic cardiomyopathy (*n* = 32) from healthy comparators (*n* = 30). The most discriminative feature was gray level non-uniformity, a radiomics texture feature representing heterogeneity. It seems intuitive that this feature would be defining of the irregular myofibrillar architecture of hypertrophic cardiomyopathy. Similar to our observations, in particular with diabetes, it appears that the observed radiomics signatures may reflect clinically meaningful information about significant tissue level changes. Furthermore, studies have demonstrated the ability of CMR radiomics to distinguish important conditions that appear morphologically similar with conventional image analysis. For instance, Neisius et al. (15) demonstrated high performance of CMR radiomics models applied to native T1 images to distinguish hypertensive heart disease (*n* = 53), hypertrophic cardiomyopathy (*n* = 108), and healthy volunteers (*n* = 71). There is also emerging work on using CMR radiomics to identify areas of myocardial infarction from non-contrast cine image (16, 33, 34) and to identify acute from chronic myocardial infarction (33).

Our paper constitutes the most comprehensive study to assess the relationship between CMR radiomics and cardiovascular risk factors. However, the concept of utilizing information from CMR to obtain more complex geometric information has been addressed previously using atlas-based shape measures. Cardiac atlases produce statistical shape models, giving highly detailed morphometric information (35–37). Directly comparable to our findings, Gilbert et al. (38) demonstrate unique morphometric variations associated with individual risk factors (high blood pressure, smoking, high cholesterol, diabetes, angina), which could be quantified and visualized on constructed atlases. The derivation of radiomics shape features is methodologically different from cardiac atlases, however there are conceptual similarities about the type of information they provide. Both seem to suggest that geometric features not captured by current image analysis approaches may be extracted from existing CMR images and that this information seems to provide additional insight into patterns of cardiac remodeling. CMR radiomics has several advantages over cardiac atlas models. The signal intensity based radiomics features (first-order, texture) have great potential for not only better disease discrimination and outcome prediction, but also gaining deeper insights into disease processes at the tissue level; such information is not provided by cardiac atlas morphometrics. CMR radiomics analysis does not require any dedicated acquisitions or post-processing and the extraction of radiomics features and model building are computationally simpler than atlas models. Therefore, there is real potential for radiomics to enter the clinical workflow as a very high yield and complementary image analysis tool.

Note that in this study we chose to select a different healthy subsample than in Petersen et al. (22). This is due to the differences in the objectives of the papers. While Petersen et al. (22) focused on the estimation of normal ranges of cardiac indices of structure and function and thus used very strict inclusion criteria, we are concerned with the study of cardiovascular risk factors and therefore we excluded subjects with known cardiovascular risk factor or disease.

## Limitations and Future Work

To the best of our knowledge, this is the largest study to assess the performance of CMR radiomics model in discriminating several important cardiovascular risk factors. Our findings demonstrate the feasibility of CMR radiomics models to identify cardiac changes related to important cardiovascular risk factors (diabetes, hypertension, high cholesterol, and smoking) with greater accuracy than conventional indices. The UKB provides an excellent platform for this study with a large sample of well characterized participants with linked CMR imaging. However, the data collection was conducted through a combination of a touchscreen questionnaire and a face-to-face nurse interview, and thus there remains some concerns about the accuracy and objectivity of the self-reported conditions. Studies with consideration of more sophisticated statistical methods to better account for confounding factors, as well as with inclusion of external validation cohorts, are needed to produce and validate more disease-specific and generalizable models. In particular, there is a need for prospective studies to determine the clinical utility of these models in providing incremental cardiovascular risk information.

As for the pipeline implemented in this paper, alternative approaches may merit exploration, such as testing different methods for feature selection [e.g., LASSO (39), combination of filter and wrapper-based methods (40)], or applying extensive hyper-parameter optimization for each risk group. Also, while cross-validation was performed in the feature selection process to reduce the instability of radiomics features, other strategies have been proposed such as prior clustering of redundant features (41), or using a concordance correlation coefficient (42). Additionally, there is need for proper evaluation of the reproducibility of radiomics features across segmentation protocols and also across imaging acquisitions, which is important due to non-standard pixel values and large variation in signal intensities (43). Wider use of radiomics quality scores (44) would also enable better quality and more uniform reporting of radiomics studies and foster research reproducibility. Finally, as a common problem of artificial intelligence-based radiomics approaches, we have not assessed the practical value of the present results since there is no comparative gold standard that can be used for comparison.

## Conclusions

CMR radiomics is an emerging technique for deeper and more accurate cardiac phenotyping in comparison to conventional image analysis. Our preliminary results based on a large sample from the UKB indicates the feasibility of CMR radiomics analysis and potential clinical utility in superior image phenotyping of major cardiovascular risk factors, including diabetes, hypertension, high cholesterol, and smoking. The clinical value of these radiomics signatures for prediction of downstream events warrants further investigation in prospective cohorts.

## Data Availability Statement

This research was conducted using the UK Biobank re-source under Application 2964. UK Biobank will make the data available to all bona fide researchers for all types of health-related research that is in the public interest, without preferential or exclusive access for any person. All researchers will be subject to the same application process and approval criteria as specified by UK Biobank. For the detailed access procedure see http://www.ukbiobank.ac.uk/register-apply/.

## Ethics Statement

The studies involving human participants were reviewed and approved by NHS National Research Ethics Service (17th June 2011, Ref11/NW/0382). The patients/participants provided their written informed consent to participate in this study.

## Author Contributions

SKP and SEP contributed to study concepts, methods, and underlying data collection. SEP, SKP, SNe, and ZR-E provided support on clinical aspects of the study. IC, ZR-E, OC, and KL drafted the manuscript. IC, KL, SNa, OC, and MG designed the machine learning methods. IC performed the data pre-processing and data analysis. All authors contributed to the article and approved the submitted version and participated in the analysis of the data, critical revision of the manuscript, and final approval of the submitted manuscript.

## Conflict of Interest

The authors declare that the research was conducted in the absence of any commercial or financial relationships that could be construed as a potential conflict of interest. The reviewer AS declared a past co-authorship with some of the authors SEP, SKP, and SNe to the handling editor

## Footnotes

^1^https://www.radiomics.io/pyradiomics.html

## Abbreviations

ACC: Accuracy
AUC: Area under the curve
bSSFP: Balanced steady state free procession
Conv: Conventional cardiovascular magnetic resonance indices
CMR: Cardiovascular magnetic resonance
CV: Cardiovascular
ECG: Electrocardiogram
ED: End-diastole
ES: End-systole
F: First-order; radiomics feature
LV: Left ventricle
LVEDV: Left ventricle end-diastolic volume
LVEDVi: Indexed left ventricle end-diastolic volume
LVEF: Left ventricle ejection fraction
LVESV: Left ventricle end-systolic volume
LVESVi: Indexed left ventricle end-systolic volume
LVM: Left ventricle mass
LVMi: Indexed left ventricle mass
LR: Logistic regression
LVSV: Left ventricle stroke volume
LVSVi: Indexed left ventricle stroke volume
ML: Machine learning
MYO: Left ventricle myocardium
RF: Random forest
Rad: Radiomics features
ROI: Region of interest
RV: Right ventricle
RVEDV: Right ventricle end-diastolic volume
RVEDVi: Indexed right ventricle end-diastolic volume
RVEF: Right ventricle ejection fraction
RVESV: Right ventricle end-systolic volume
RVESVi: Indexed right ventricle end-systolic volume
RVSV: Right ventricle stroke volume
RVSVi: Indexed right ventricle stroke volume
S: Shape-based radiomics features
SFFS: Sequential forward feature selection
SVM: Support vector machines
T: Texture-based radiomics features
TE: Echo time
TR: Repetition time
UKB: UK Biobank.

## References

[1] Zhao F Zhang H Wahle A Thomas MT Stolpen AH Scholz TD. Congenital aortic disease: 4D magnetic resonance segmentation and quantitative analysis. Med Image Anal. (2009) 13:483–93. 10.1016/j.media.2009.02.005 19303351 PMC2727644

[2] Suinesiaputra A Frangi AF Kaandorp TA Lamb HJ Bax JJ Reiber JH. Automated detection of regional wall motion abnormalities based on a statistical model applied to multislice short-axis cardiac MR images. IEEE Trans Med Imaging. (2009) 28:595–607. 10.1109/TMI.2008.2008966 19211347

[3] Suinesiaputra A Ablin P Alba X Alessandrini M Allen J Bai W. Statistical shape modeling of the left ventricle: myocardial infarct classification challenge. IEEE J Biomed Health Inform. (2018) 22:503–515. 10.1109/JBHI.2017.2652449 28103561 PMC5857476

[4] Lekadir K Albà X Pereañez M Frangi AF Statistical shape modeling using partial least squares: application to the assessment of myocardial infarction. In: Camara O. Mansi T Pop M Rhode K Sermesant M Young A, editors. Statistical Atlases and Computational Models of the Heart. Imaging and Modelling Challenges. STACOM 2015. Lecture Notes in Computer Science. Cham: Springer (2016). p. 130–9. 10.1007/978-3-319-28712-6_14

[5] Petersen SE Sanghvi MM Aung N Cooper JA Paiva JM Zemrak F. The impact of cardiovascular risk factors on cardiac structure and function: Insights from the UK Biobank imaging enhancement study. PLoS ONE. (2017) 12:e0185114. 10.1371/journal.pone.0185114 28973022 PMC5626035

[6] Raisi-Estabragh Z Izquierdo C Campello VM Martin-Isla C Jaggi A Harvey NC. Cardiac magnetic resonance radiomics: basic principles and clinical perspectives. Eur Heart J Cardiovasc Imaging. (2020) 21:349–56. 10.1093/ehjci/jeaa028 32142107 PMC7082724

[7] Martin-Isla C Campello VM Izquierdo C Raisi-Estabragh Z Baeßler B Petersen SE. Image-based cardiac diagnosis with machine learning: a review. Front Cardiovasc Med. (2020) 7:1. 10.3389/fcvm.2020.00001 32039241 PMC6992607

[8] Aerts H Velazquez E Leijenaar R. Decoding tumour phenotype by noninvasive imaging using a quantitative radiomics approach. Nat Commun. (2014) 5:4006. 10.1038/ncomms5006 24892406 PMC4059926

[9] Aerts H. The potential of radiomic-based phenotyping in precision medicine: a review. JAMA Oncol. (2016) 2:1636–42. 10.1001/jamaoncol.2016.2631 27541161

[10] Lambin P Leijenaar RTH Deist TM Peerlings J de Jong EEC van Timmeren J. Radiomics: the bridge between medical imaging and personalized medicine. Nat Rev Clin Oncol. (2017) 14:749–62. 10.1038/nrclinonc.2017.141 28975929

[11] Coroller TP Grossmann P Hou Y Rios Velazquez E Leijenaar RT Hermann G. CT-based radiomic signature predicts distant metastasis in lung adenocarcinoma. Radiother Oncol. (2015) 114:345–50. 10.1016/j.radonc.2015.02.015 25746350 PMC4400248

[12] Gillies RJ Kinahan PE Hricak H. Radiomics: images are more than pictures, they are data. Radiology. (2016) 278:563–77. 10.1148/radiol.2015151169 26579733 PMC4734157

[13] Napel S Mu W Jardim-Perassi BV Aerts HJWL Gillies RJ. Quantitative imaging of cancer in the postgenomic era: Radio(geno)mics, deep learning, and habitats. Cancer. (2018) 124:4633–49. 10.1002/cncr.31630 30383900 PMC6482447

[14] Chen CH Chang CK Tu CY Liao WC Wu BR Chou KT. Radiomic features analysis in computed tomography images of lung nodule classification. PLoS ONE. (2018) 13:e0192002. 10.1371/journal.pone.0192002 29401463 PMC5798832

[15] Neisius U El-Rewaidy H Nakamori S Rodriguez J Manning WJ Nezafat R. Radiomic analysis of myocardial native t1 imaging discriminates between hypertensive heart disease and hypertrophic cardiomyopathy. JACC Cardiovasc Imaging. (2019) 12:1946–54. 10.1016/j.jcmg.2018.11.024 30660549 PMC7032053

[16] Larroza A López-Lereu MP Monmeneu JV Gavara J Chorro FJ Bodí V. Texture analysis of cardiac cine magnetic resonance imaging to detect nonviable segments in patients with chronic myocardial infarction. Med Phys. (2018) 45:1471–80. 10.1002/mp.12783 29389013

[17] Baessler B Luecke C Lurz J Klingel K von Roeder M de Waha S. Cardiac MRI texture analysis of T1 and T2 maps in patients with infarctlike acute myocarditis. Radiology. (2018) 289:357–65. 10.1148/radiol.2018180411 30084736

[18] Cetin I Sanroma G Petersen SE Napel S Camara O Gonzalez Ballester MA A radiomics approach to computer-aided diagnosis with cardiac cine-MRI. In: Pop M, editor. Statistical Atlases and Computational Models of the Heart. ACDC and MMWHS Challenges. STACOM Lecture Notes in Computer Science. Cham: Springer (2018). p. 10663 10.1007/978-3-319-75541-0_9

[19] Raisi-Estabragh Z Petersen SE. Cardiovascular research highlights from the UK Biobank: opportunities and challenges. Cardiovasc Res. (2020) 116:e12–e5. 10.1093/cvr/cvz294 31778147

[20] Sudlow C Gallacher J Allen N Beral V Burton P Danesh J. UK biobank: an open access resource for identifying the causes of a wide range of complex diseases of middle and old age. PLoS Med. (2015) 12:e1001779. 10.1371/journal.pmed.1001779 25826379 PMC4380465

[21] Petersen SE Matthews PM Francis JM Robson MD Zemrak F Boubertakh R. UK Biobank's cardiovascular magnetic resonance protocol. J Cardiovasc Magn Reson. (2016) 18:8. 10.1186/s12968-016-0227-4 26830817 PMC4736703

[22] Petersen SE Aung N Sanghvi MM Zemrak F Fung K Paiva JM. Reference ranges for cardiac structure and function using cardiovascular magnetic resonance (CMR) in Caucasians from the UK biobank population cohort. J Cardiovasc Magn Reson. (2017) 19:18. 10.1186/s12968-017-0327-9 28178995 PMC5304550

[23] van Griethuysen JJM Fedorov A Parmar C Hosny A Aucoin N Narayan V. Computational radiomics system to decode the radiographic phenotype. Cancer Res. (2017) 77:e104–e7. 10.1158/0008-5472.CAN-17-0339 29092951 PMC5672828

[24] Shao XN Sun YJ Xiao KT Zhang Y Zhang WB Kou ZF. Texture analysis of magnetic resonance T1 mapping with dilated cardiomyopathy: A machine learning approach. Medicine. (2018) 97:e12246. 10.1097/MD.0000000000012246 30212958 PMC6156048

[25] Schofield R Ganeshan B Fontana M Nasis A Castelletti S Rosmini S. Texture analysis of cardiovascular magnetic resonance cine images differentiates aetiologies of left ventricular hypertrophy. Clin Radiol. (2019) 74:140–9. 10.1016/j.crad.2018.09.016 30527518

[26] Cetin I Petersen SE Napel S Camara O Gonzalez Ballester MA Lekadir K A radiomics approach to analyze cardiac alterations in hypertension. In: 2019 IEEE 16th International Symposium on Biomedical Imaging (ISBI 2019), Venice (2019). p. 640–3. 10.1109/ISBI.2019.8759440

[27] Sebastian R MLxtend: providing machine learning data science utilities extensions to Python's scientific computing stack. J. Open Source Softw. (2018) 3:638 10.21105/joss.00638

[28] Pedregosa F Varoquaux G Gramfort A Michel V Thirion B Grisel O Scikit-learn: machine learning in python. JMLR. (2011) 12:2825–30.

[29] Looney SW A statistical technique for comparing the accuracies of several classifiers. Pattern Recogn Lett. (1988) 8:5-9. 10.1016/0167-8655(88)90016-5

[30] Cochran WG. The comparison of percentages in matched samples. Biometrika. (1950) 37:256–66. 10.1093/biomet/37.3-4.256 14801052

[31] Baeßler B Mannil M Maintz D Alkadhi H Manka R. Texture analysis and machine learning of non-contrast T1-weighted MR images in patients with hypertrophic cardiomyopathy-Preliminary results. Eur J Radiol. (2018) 102:61–7. 10.1016/j.ejrad.2018.03.013 29685546

[32] Nadruz W Jr Claggett B Gonçalves A Querejeta-Roca G Fernandes-Silva MM Shah AM. Smoking and cardiac structure and function in the elderly: the ARIC Study (Atherosclerosis Risk in Communities). Circ Cardiovasc Imaging. (2016) 9:e004950. 10.1161/CIRCIMAGING.116.004950 27625349 PMC5193104

[33] Larroza A Materka A López-Lereu MP Monmeneu JV Bodí V Moratal D. Differentiation between acute and chronic myocardial infarction by means of texture analysis of late gadolinium enhancement and cine cardiac magnetic resonance imaging. Eur J Radiol. (2017) 92:78–83. 10.1016/j.ejrad.2017.04.024 28624024

[34] Baessler B Mannil M Oebel S Maintz D Alkadhi H Manka R. Subacute and chronic left ventricular myocardial scar: accuracy of texture analysis on nonenhanced cine MR images. Radiology. (2018) 286:103–12. 10.1148/radiol.2017170213 28836886

[35] Medrano-Gracia P Cowan BR Ambale-Venkatesh B Bluemke DA Eng J Finn JP. Left ventricular shape variation in asymptomatic populations: the Multi-Ethnic Study of Atherosclerosis. J Cardiovasc Magn Reson. (2014) 16:56. 10.1186/s12968-014-0056-2 25160814 PMC4145340

[36] Bai W Shi W de Marvao A Dawes TJ O'Regan DP Cook SA. A bi-ventricular cardiac atlas built from 1000+ high resolution MR images of healthy subjects and an analysis of shape and motion. Med Image Anal. (2015) 26:133–45. 10.1016/j.media.2015.08.009 26387054

[37] Young AA Frangi AF. Computational cardiac atlases: from patient to population and back. Exp Physiol. (2009) 94:578–96. 10.1113/expphysiol.2008.044081 19098087

[38] Gilbert K Bai W Mauger C. Independent left ventricular morphometric atlases show consistent relationships with cardiovascular risk factors: a UK biobank study. Sci Rep. (2019) 9:1130. 10.1038/s41598-018-37916-6 30718635 PMC6362245

[39] Lee SH Cho HH Lee HY Park H. Clinical impact of variability on CT radiomics and suggestions for suitable feature selection: a focus on lung cancer. Cancer Imag. (2019) 19:54. 10.1186/s40644-019-0239-z 31349872 PMC6660971

[40] Shakir H Deng Y Rasheed H. Radiomics based likelihood functions for cancer diagnosis. Sci Rep. (2019) 9:9501. 10.1038/s41598-019-45053-x 31263186 PMC6603029

[41] Lecler A Duron L Balvay D. Combining multiple magnetic resonance imaging sequences provides independent reproducible radiomics features. Sci Rep. (2019) 9:2068. 10.1038/s41598-018-37984-8 30765732 PMC6376058

[42] Peerlings J Woodruff HC Winfield JM. Stability of radiomics features in apparent diffusion coefficient maps from a multi-centre test-retest trial. Sci Rep. (2019) 9:4800. 10.1038/s41598-019-41344-5 30886309 PMC6423042

[43] Park JE Park SY Kim HJ Kim HS. Reproducibility and generalizability in radiomics modeling: possible strategies in radiologic and statistical perspectives. Korean J Radiol. (2019) 20:1124–37. 10.3348/kjr.2018.0070 31270976 PMC6609433

[44] Park JE Kim D Kim HS Park SY Kim JY Cho SJ. Quality of science and reporting of radiomics in oncologic studies: room for improvement according to radiomics quality score and TRIPOD statement. Eur Radiol. (2020) 30:523–36. 10.1007/s00330-019-06360-z 31350588

