# Supplementary material for: Radiomics Signatures of Cardiovascular Risk Factors in Cardiac MRI: Results From the UK Biobank
Source: Front Cardiovasc Med. 2020 Nov 2;7:591368. doi: 10.3389/fcvm.2020.591368 (PMC7667130; doi:10.3389/fcvm.2020.591368)
Supplement: Supplementary file 1 [file Data_Sheet_1.pdf]

Supplementary Table

1 EXTRACTED RADIOMICS FEATURES

**Shape Features**	
Feature name	Interpretation
Volume	The volume of the ROI is approximated by multiplying the number of voxels in the ROI by the volume of a single voxel.
Surface Area	Surface Area is an approximation of the ROI surface based on triangulation interpretation.
Surface Area to Volume ratio	For details refer to preceding 2 features. Lower values of this parameter indicate a sphere-like shape of the ROI.
Sphericity	A measure of the roundness of the ROI relative to a sphere.
Compactness1	A measure of how compact the shape of the ROI is relative to a sphere.
Compactness2	A measure of how compact the shape of the ROI is relative to a sphere.
Spherical Disproportion	The inverse of Sphericity. Measures the ratio of the surface area of the ROI to the surface area of a sphere with the same volume as the ROI.
Maximum 3D diameter	The largest pairwise Euclidean distance between ROI surface voxels.
Maximum 2D diameter (Slice)	The largest pairwise Euclidean distance between ROI surface voxels of specific axial slice.
Maximum 2D diameter (Column)	The largest pairwise Euclidean distance between ROI surface voxels of specific coronal slice.
Maximum 2D diameter (Row)	The largest pairwise Euclidean distance between ROI surface voxels of specific sagittal slice.
Major Axis	A feature derived from the principal component analysis proportional to the square root of length of the largest principal component axes
Minor Axis	A feature derived from the principal component analysis proportional to the square root of length of the second largest principal component axes.

Least Axis	A feature derived from the principal component analysis proportional to the square root of length of the second largest principal component axes.
Elongation	A feature derived from the principal component analysis proportional to the ratio of lengths of the second largest and the largest principal component axes.
Flatness	A feature derived from the principal component analysis proportional to the ratio of lengths of the smallest and the largest principal component axes.
**First Order Features**	
Energy	Energy is a measure of the magnitude of voxel values in an image.
Total Energy	Total Energy is the value of Energy feature scaled by the volume of the voxel in cubic mm.
Entropy	Entropy specifies the uncertainty or randomness in the image values. It measures the average amount of information required to encode the image values.
Minimum	Minimum intensity value present in the ROI.
10th percentile	Value below which 10% of the intensities may be found in the histogram of the ROI.
90th percentile	Value below which 90% of the intensities may be found in the histogram of the ROI.
Maximum	Maximum grey level intensity found in the ROI.
Mean	Mean gray level intensity found in the ROI.
Median	Median grey level intensity found in the ROI.
Interquartile Range	The difference between the 25th and 75th percentile of ROI.
Range	A difference between the maximum and minimum gray tone present in the ROI.
Mean Absolute Deviation	MAD is the mean distance of all intensity values from the Mean Value present in the ROI.
Robust Mean Absolute Deviation	Robust MAD is a modification of MAD which takes into account only ROI intensities present in between 10th and 90th percentile which helps to avoid noise impact.
Root Mean Squared	RMS is the square-root of the mean of all the intensity values squared. Characterizes the magnitude of the image gray tone.

Standard Deviation	Measures the amount of variation from the mean intensity value.
Skewness	Skewness measures the asymmetry of the distribution of values around the Mean value
Kurtosis	Kurtosis measures the ‘peakedness’ of the values distribution in the image ROI.
Variance	Variance is the the mean of the squared distances of each intensity value from the Mean value.
Uniformity	Uniformity is a measure of the sum of the squares of each intensity value. This is a measure of the heterogeneity of the ROI.
**Texture Features** Gray level co-occurence matrix (GLCM)	
Autocorrelation	Autocorrelation detects repetitive patterns present in the ROI. Intends to measure the magnitude of the fineness and coarseness of texture .
Joint Average	Returns the mean gray level intensity of the i distribution.
Cluster Prominence	Cluster Prominence is a measure of the skewness and asymmetry of the GLCM.
Cluster Shade	Cluster Shade is a measure of the skewness and uniformity of the GLCM.
Cluster Tendency	Cluster Tendency is a measure of groupings of voxels within the ROI with similar gray-level values.
Contrast	Contrast is a measure of the local intensity variation, favoring values away from the diagonal of the GLCM.
Correlation	Correlation is a value between 0 (uncorrelated) and 1 (perfectly correlated) showing the linear dependency of gray level values to their respective voxels in the GLCM.
Difference Average	Difference Average measures the relationship between occurrences of pairs with similar intensity values and occurrences of pairs with differing intensity values in GLCM.
Difference Entropy	Difference Entropy is a measure of the randomness/variability in neighborhood intensity value differences.
Difference Variance	Difference Variance is a measure of heterogeneity that places higher weights on differing intensity level pairs that deviate more from the mean.

Dissimilarity	Mathematically equal to Difference Average
Joint Energy	Energy is a measure of how homogeneous are the patterns in the ROI.
Joint Entropy	Joint entropy is a measure of the randomness/variability in neighborhood intensity values.
Correlation1	Alternative definition of Correlation based on ratio of entropy dependencies to the maximum entropy.
Correlation2	Alternative definition of Correlation based on entropy dependencies. Uses square root of entropies difference instead of the max.
Inverse Difference Moment (IDM)	IDM is a measure of the local homogeneity of an image.
Inverse Difference Moment Normalized (IDMN)	Normalization of IDM. IDMN normalizes the square of the difference between neighboring intensity values by dividing over the square of the total number of discrete intensity values.
Inverse Difference (ID)	ID is another measure of the local homogeneity of an image.
Inverse Difference Normalized (IDN)	IDN normalizes the difference between the neighboring intensity values by dividing over the total number of discrete intensity values.
Inverse Variance	Inverse of the variance. Sums up the elements of the GLCM matrix while decreasing the values which lay further from the diagonal proportional to the distance.
Maximum Probability	Maximum Probability is the occurrence of the most predominant pair of neighboring intensity values.
Sum Average	Sum Average measures the relationship between occurrences of pairs with lower intensity values and occurrences of pairs with higher intensity values.
Sum Entropy	Sum Entropy is a sum of neighborhood intensity value differences.
Sum of Squares	Sum of Squares is a measure in the distribution of neighboring intensity level pairs about the mean intensity level in the GLCM.
Homogeneity1	An alternative measure of the local homogeneity of an image.
Homogeneity2	An alternative measure of the local homogeneity of an image.
Gray level size zone matrix (GLSZM)	

Small area emphasis (SAE)	SAE measures how many small regions with the same intensity value(fine texture) are present in the ROI opposed to big regions with same intensity value(homogeneous texture).
Large Area emphasis(LAE)	LAE measures how many big regions with same intensity value(homogeneous texture) are present in the ROI opposed to the small regions with the same intensity value(fine texture).
Gray Level Non-Uniformity (GLN)	GLN measures the variability of gray-level intensity values in the image, with a lower value indicating more homogeneity in intensity values and higher value indicating the presence of fine texture texture.
Gray Level Non-Uniformity Normalized (GLNN)	Normalized version of GLN which takes into account the number of zones with the same intensity present within the ROI.
Size-Zone Non-Uniformity (SZN)	SZN measures the variability of the size zone volumes(regions with the same intensity) in the image, with a lower value indicating that ROI has even size zones volumes.
Size-Zone Non-Uniformity Normalized (SZNN)	Normalized SZN which takes into account the number of zones with the same intensity present within the ROI.
Zone Percentage (ZP)	ZP measures the coarseness of the texture by taking the ratio of number of zones with the same intensity and number of voxels in the ROI.
Gray Level Variance (GLV)	GLV measures the variance in gray level intensities for the zones (regions with same intensity).
Zone Variance (ZV)	ZV measures the variance in zone(region with the same intensity) size .
Zone Entropy (ZE)	ZE measures the uncertainty/randomness in the distribution of zone sizes and gray levels.
Low Gray Level Zone Emphasis (LGLZE)	LGLZE measures the distribution of lower gray-level size zones, with a higher value indicating a greater proportion of lower gray-level values and size zones in the image.
High Gray Level Zone Emphasis (HGLZE)	HGLZE measures the distribution of the higher gray-level values, with a higher value indicating a greater proportion of both higher gray-level values and size zones in the image.
Small area low gray level emphasis (SALGLE)	SALGLE measures the proportion in the image of the joint distribution of smaller size zones with lower gray-level values.
Small area high gray level emphasis (SAHGLE)	SAHGLE measures the proportion in the image of the joint distribution of smaller size zones with higher gray-level values.

Large area low gray level emphasis (LALGLE)	LALGLE measures the proportion in the image of the joint distribution of larger size zones with lower gray-level values.
Large area high gray level emphasis (LAHGLE)	LAHGLE measures the proportion in the image of the joint distribution of larger size zones with higher gray-level values.
Gray level run length matrix (GLRLM)	
Short run emphasis (SRE)	SRE is a measure of the distribution of short run lengths, with a greater value indicative of shorter run lengths and more fine textural textures.
Long run emphasis (LRE)	LRE is a measure of the distribution of long run lengths, with a greater value indicative of longer run lengths and more coarse structural textures.
Gray level non-uniformity (GLN)	GLN measures the similarity of gray-level intensity values in the image, where a lower GLN value correlates with a greater similarity in intensity values.
Gray level non-uniformity normalized (GLNN)	GLNN measures the similarity of gray-level intensity values in the image, where a lower GLNN value correlates with a greater similarity in intensity values. This is the normalized version of the GLN formula.
Run length non-uniformity (RLN)	RLN measures the similarity of run lengths throughout the image, with a lower value indicating more homogeneity among run lengths in the image.
Run length non-uniformity normalized (RLNN)	RLNN measures the similarity of run lengths throughout the image, with a lower value indicating more homogeneity among run lengths in the image. This is the normalized version of the RLN formula.
Run percentage (RP)	RP measures the coarseness of the texture by taking the ratio of number of runs and number of voxels in the ROI.
Gray level variance (GLV)	GLV measures the variance in gray level intensity for the runs.
Run variance (RV)	RV is a measure of the variance in runs for the run lengths.
Run entropy (RE)	RE measures the uncertainty/randomness in the distribution of run lengths and gray levels. A higher value indicates more heterogeneity in the texture patterns.
Low gray level run emphasis (LGLRE)	LGLRE measures the distribution of low gray-level values, with a higher value indicating a greater concentration of low gray-level values in the image.

High gray level run emphasis (HGLRE)	HGLRE measures the distribution of the higher gray-level values, with a higher value indicating a greater concentration of high gray-level values in the image.
Short run low gray level emphasis (SRLGLE)	SRLGLE measures the joint distribution of shorter run lengths with lower gray-level values.
Short run high gray level emphasis (SRHGLE)	SRHGLE measures the joint distribution of shorter run lengths with higher gray-level values.
Long run low gray level emphasis (LRLGLE)	LRLGLE measures the joint distribution of long run lengths with higher gray-level values.
Long run high gray level emphasis (LRHGLE)	LRHGLE measures the proportion in the image of the joint distribution of larger size zones with higher gray-level values.
Neigbouring Gray Tone Difference Matrix (NGTDM)	
Coarseness	Coarseness is a measure of average difference between the center voxel and its neighbourhood and is an indication of the spatial rate of change. A higher value indicates a lower spatial change rate and a locally more uniform texture.
Contrast	Contrast is a measure of the spatial intensity change, but is also dependent on the overall gray level dynamic range. Contrast is high when both the dynamic range and the spatial change rate are high, i.e. an image with a large range of gray levels, with large changes between voxels and their neighborhood.
Busyness	A measure of the change from a pixel to its neighbor. A high value for busyness indicates a busy image, with rapid changes of intensity between pixels and its neighborhood.
Complexity	An image is considered complex when there are many primitive components in the image, i.e. the image is non-uniform and there are many rapid changes in gray level intensity.
Strength	Strength is a measure of the primitives in an image. Its value is high when the primitives are easily defined and visible, i.e. an image with slow change in intensity but more large coarse differences in gray level intensities.
Gray level dependence matrix (GLDM)	
Small dependence emphasis (SDE)	Measures how many small dependencies are present in ROI. Greater values represents smaller dependence and less homogeneous texture

Large dependence emphasis (LDE)	Measures how many large dependencies are present in ROI. Greater value indicates larger dependence and more homogeneous texture.
Gray level non-uniformity (GLN)	Measures the similarity of gray-level intensity values in the image. Higher value indicates smaller similarity whereas lower value indicates higher similarity in gray level intensity values.
Gray level non-uniformity normalized (GLNN)	GLNN measures the similarity of gray-level intensity values in the image, where a lower GLNN value correlates with a greater similarity in intensity values. This is the normalized version of the GLN formula.
Dependence non-uniformity (DN)	Measures the similarity of dependence throughout the image, with a lower value indicating more homogeneity among dependencies in the image.
Dependence non-uniformity normalized (DNN)	Measures the similarity of dependence in the image, with a lower value indicating more homogeneity among dependencies in the image. This is the normalized version of the DLN formula.
Gray level variance (GLV)	Measures the variance in grey level in the image.
Dependence variance (DV)	Measures the variance in gray level dependence size in the image.
Dependence entropy (DE)	DE measures the randomness in the gray level dependencies and gray levels.
Dependence percentage (DP)	DP is the ratio between voxels with a dependence zone and the total number of voxels in the image.
Low gray level emphasis (LGLE)	Measures the distribution of low gray-level values, with a higher value indicating a greater concentration of low gray-level values in the image.
High gray level emphasis (HGLE)	Measures the distribution of the higher gray-level values, with a higher value indicating a greater concentration of high gray-level values in the image.
Small dependence low gray level emphasis (SDLGLE)	Measures the joint distribution of small dependence with lower gray-level values.
Small dependence high gray level emphasis (SDHGLE)	Measures the joint distribution of small dependence with higher gray-level values.
Large Dependence Low Gray Level Emphasis (LDLGLE)	Measures the joint distribution of large dependence with lower gray-level values.
Large Dependence High Gray Level Emphasis (LDHGLE)	Measures the joint distribution of large dependence with higher gray-level values.

REFERENCES

[1]Pyradiomics Documentation,
http://pyradiomics.readthedocs.io/en/latest/index.html

[2]van Griethuysen, Joost JM, et al. "Computational Radiomics System to Decode the Radiographic Phenotype." Cancer research 77.21 (2017): e104-e107.

[3]Zwanenburg, Alex, et al. "Image biomarker standardisation initiative-feature definitions." arXiv preprint arXiv:1612.07003 (2016).

[4]Cetin I, Sanroma G, Petersen SE, Napel S, Camara O, Gonzalez Ballester MA, Lekadir K. A radiomics approach to computer-aided diagnosis with cardiac cine-MRI. In: Pop M, editors. Statistical Atlases and Computational Models of the Heart. ACDC and MMWHS Challenges. STACOM Lecture Notes in Computer Science. Cham: Springer (2018). p. 10663. doi: 10.1007/978-3-319-75541-0_9
