# Supplementary material for: Radiomics Signatures of Cardiovascular Risk Factors in Cardiac MRI: Results From the UK Biobank
Source: Front Cardiovasc Med. 2020 Nov 2;7:591368. doi: 10.3389/fcvm.2020.591368 (PMC7667130; doi:10.3389/fcvm.2020.591368)
Supplement: Supplementary file 2 [file Data_Sheet_2.pdf]

Supplementary Material

1 Radiomics feature selection and model building

After radiomics feature extraction, the next step consisted of identifying the combination of features that best discriminate the no-risk vs. at-risk subgroups for all risk factors. In this case, the selected radiomics features would encode alterations due to the risk factors under investigation. For this purpose, machine learning (ML) techniques (support vector machines, SVM; random forests, RF; logistic regression, LR) were implemented in combination with a feature selection algorithm. Implementation of the SFFS and the ML techniques was based on the mlxtend (version 0.17.0) and scikit-learn (version 0.20.3) python-based libraries, respectively. An optimization process was performed by tuning the hyper-parameters of the ML techniques to find the optimal approach for the discrimination tasks. In total 33 combinations of ML methods and hyper-parameter values were tested:

- SVM (15 configurations): linear vs radial-basis function (RBF) kernel, gamma parameter of the RBF kernel (values of 0.1, 1 and 10) and regularization parameter (C, with values 0.1, 1 and 10);
- LR (6 configurations): l1 (liblinear library [1]) vs l2 (lbfgs library [2]) penalty regularization and regularization parameter (C, with values 0.1, 1 and 10);
- RF (12 configurations): number of trees/estimators in the forest (nest with values of 10 and 100), maximum number of features in the best split (maxfeat = none, i.e. taking all features; maxfeat = sqrt, i.e. taking the square root of the number of features; maxfeat = log2, i.e. taking log2 of the number of features) and split quality criterion (gini impurity vs entropy).

The selected radiomics features resulted from the SFFS algorithm and ML techniques were combined to create the radiomics signature that best encode the changes in CMR induced by the different cardiovascular risk factors.

Table 1: Selected radiomics features and prediction performance for the optimal machine learning technique configurations.

Classifier	S	F	T	W	ACC	AUC
**SVM**	1	2	4	1	0.763	0.770
**LR**	5	3	3	0	0.782	0.803
**RF**	2	2	5	1	0.761	0.791

SVM: Support vector machines, LR: logistic regression, RF: random forests, S: shape, F: first-order, T: texture, W: size, ACC: accuracy, AUC: area under the curve

1.1 Hyper-parameter optimization on a subset of the data

To illustrate the process of hyperparameter optimization, we compared variants of the three studied ML techniques (SVM, RF, LR) on the subset of the data composed of diabetes vs. healthy controls (on 243x2 cases), generating a total of 33 different combinations of methods and hyper-parameter values.

The best discriminative performances for each ML technique were of 0.763 (SVM), 0.782 (LR) and 0.761 (RF), as can be seen in Table 1. These results were obtained with different amount (8, 11 and 10 features, respectively) and distribution of radiomics features. Notably, the best prediction performance in this data subset was provided by the LR technique, which selected 5 shape, 3 first-order and 3 texture based radiomic features.

Table 2 shows the results of two phases of the Cochran's Q statistical tests, aiming at first identifying the best hyper-parameter combinations within each ML technique separately and secondly comparing the different ML techniques among them. In a first step, statistically significant differences were found for the different combinations of the LR and RF techniques but the null hypothesis was accepted for SVM. Subsequently, 9 classifiers were implemented with different ML techniques and hyperparameters for the next test; 2 SVM (C1, C2), 2 LR (C3, C4) and 5 RF (C5, C6, C7, C8, C9) classifiers. A statistical test was performed on all the classifiers and a p-value less than 0.5 was obtained, showing that there was a statistical difference among them. Afterwards a Bonferroni corrected post-hoc test was employed, with the new p-value equal to 0.0014, to perform pairwise comparisons. As a result of this test, statistical significant differences were found when comparing five different ML techniques (C1, C2, C3, C4 and C9), as illustrated in Table 2. After considering the overall prediction performances of these selected classifiers and the pairwise comparison results in Table 2, the optimal LR classifier, i.e. C4, was selected as the best method overall..

Table 2: Results of the Cochran's Q test and Bonferroni corrected McNemar post-hoc analysis. The results of the pair-wise tests show the misclassified ratios of the respective machine learning techniques.

**Classifier**	**Cochran's Q test results**			**Post-hoc**	
	**Q**	**p-value**	**Result**	**Bonferroni corrected p-value**	**Selected classifiers**
**SVM**	11.97	p=0.6	H0 accepted	-	Best AUC:<br>C1 = SVM (RBF, gamma = 0.1, C = 10)<br>Best ACC :

					C2: SVM (linear, C = 1)
**LR**	19.37	p<0.05	H0 rejected	0.03	C3: LR (l1, C = 0.1)<br><br>C4: LR (l1, C = 10) (best AUC and ACC)
**RF**	45.09	p<0.05	H0 rejected	0.0008	C5: RF (nest = 100, maxfeat = sqrt, gini)(best AUC and ACC)<br><br>C6: RF (nest = 100, maxfeat = log2, gini)<br><br>C7: RF (nest = 100, maxfeat= none, gini)<br><br>C8: RF (nest = 100, maxfeat = none, entropy)<br><br>C9: RF (nest = 10, maxfeat = none, entropy)
**Second test**					
	38.32	p<0.05	H0 rejected	0.0015	C1, C2, C3,C4 and C9
**Identified pairwise comparisons**					
1. C1 vs C9: C1 is better (with 39:85 ratio)<br><br>2. C2 vs C9: C2 is better (with 46:93 ratio)<br><br>3. C3 vs C4: C4 is better (with 29:65 ratio)<br><br>4. C4 vs C9: C4 is better (with 44:100 ratio)					

SVM: support vector machines, LR: logistic regression, RF: random forest, C: regularization parameter, RBF: radial basis functions kernel, nest: number of estimators in RF, maxfeat: maximum number of features, AUC: area under the curve, ACC: accuracy.

References

[1] R. E. Fan, K. W. Chang, C. J. Hsieh, X. R. Wang, and C. J. Lin, “LIBLINEAR: A library for large linear classification,” *J. Mach. Learn. Res.*, 2008.

[2] R. H. Byrd, P. Lu, J. Nocedal, and C. Zhu, “A Limited Memory Algorithm for Bound Constrained Optimization,” *SIAM J. Sci. Comput.*, 1995.
